# Deep-Inspiration Breath-Hold Strategy Improves Image Quality of Transthoracic Lateral Shoulder Radiography

**DOI:** 10.3390/diagnostics16162651

**Published:** 2026-08-20

**Authors:** Peng-Wei Shu, Qi-Guang Cheng, Yi Wang, Pei-Yan Feng, Yu-Hong Shen, Peng Sun, Zi-Qiao Lei, Qing Fu

**Affiliations:** 1 Department of Radiology, Union Hospital, Tongji Medical College, Huazhong University of Science and Technology, Wuhan 430022, China; spw@hust.edu.cn (P.-W.S.); daisuke14164@gmail.com (Q.-G.C.); f8v8o1bs1boy@163.com (Y.W.); 16608672334@163.com (P.-Y.F.); 13980594821@163.com (Y.-H.S.); 2 Hubei Provincial Clinical Research Center for Precision Radiology & Interventional Medicine, Wuhan 430022, China; 3 Hubei Key Laboratory of Molecular Imaging, Wuhan 430022, China; 4 Institute of Research and Clinical Innovations, Neusoft Medical Systems Co., Ltd., Shenyang 110167, China; sunpeng@neusoftmedical.com

**Keywords:** breath-hold, transthoracic lateral shoulder X-ray, image quality, BMI, traumatic shoulder injury

## Abstract

**Background/Objectives:** Transthoracic lateral shoulder radiography serves as an important method for traumatic shoulder injuries and dislocations, but tissue overlap and breathing motion often compromise image quality and are particularly prominent in obese patients. This study aims to compare the image quality differences between the deep-inspiration breath-hold (DIBH) strategy and the routine free-breathing (FB) method in transthoracic lateral shoulder radiography for patients with different body mass indices (BMI), and to clarify the clinical application value of the breath-holding strategy. **Methods:** A total of 128 patients with traumatic shoulder injuries, glenohumeral dislocation, postoperative follow-up, and symptomatic degenerative shoulder osseous lesions were prospectively enrolled to undergo radiography. Anteroposterior (AP) projection and transthoracic lateral views applying both FB and DIBH methods were performed. They were stratified into three groups (BMI < 24 kg/m^2^, 24–28 kg/m^2^, and ≥28 kg/m^2^). Two experienced radiologists independently evaluated the subjective image quality regarding anatomical depiction and abnormality visualization between the two methods, which were compared using a four-point scale (1, poor; 4, excellent), with inter-observer consistency analyzed. The edge sharpness (ES) was also calculated and compared quantitatively. The interaction effect of BMI grouping and respiratory mode regarding ES measurement was evaluated; cumulative-link mixed-effects models with subject-level random intercepts were also fitted to test whether the benefit of DIBH varied with BMI grouping for the subjective scores. **Results:** The DIBH method presented superior anatomical depiction scores relative to the FB method (*p* < 0.001). For reader 1, median scores were 3.0 (interquartile range, IQR, 3.0–3.0) for FB radiographs and 3.5 (IQR, 3.0–4.0) for the DIBH method (z = −8.014, *p* < 0.001). Consistently higher median scores were also documented by reader 2 for the DIBH method: 3.0 (IQR, 3.0–3.0) for the FB method versus 3.0 (IQR, 3.0–4.0) for the DIBH method (z = −7.616, *p* < 0.001). For lesion depiction, the scores of the DIBH images were better than those of the FB images with *p* < 0.05 for reader 1 (*p* = 0.005) and reader 2 (*p* = 0.046). Moderate-to-excellent inter-reader consistency was observed. The mean ES of the DIBH method was significantly higher than that of the FB method (2.27 ± 0.73 vs. 2.02 ± 0.70; paired t = 4.80, df = 127, *p* < 0.001). No significant differences in ES values or subjective scores for DIBH images existed among three BMI subgroups (*p* > 0.05); FB anatomical scores differed significantly for both readers (reader 1: *p* = 0.041; reader 2: *p* = 0.026). There was no significant interaction between the BMI grouping and the respiratory mode regarding ES measurement (F = 0.061, df = 2, 125, *p* = 0.941). **Conclusions:** The deep-inspiration breath-hold strategy significantly improves image quality and lesion conspicuity for depicting shoulder abnormalities compared with the routine free-breathing method in transthoracic lateral shoulder radiography, without requiring additional equipment or changes to the radiographic system, when interpreted in conjunction with the corresponding AP radiograph in clinical practice for cooperative patients.

## 1. Introduction

Shoulder radiography remains the first-line imaging modality of choice for the initial evaluation of shoulder disorders, particularly for patients with acute trauma, shoulder pain, suspected fracture, or joint dislocation [1]. Given several advantages, including wide availability, low cost, rapid acquisition, and ability to depict osseous alignment, joint congruity, and major traumatic lesions, conventional shoulder radiography plays an essential role in clinical decision-making before further imaging modalities, such as ultrasound, MRI, or CT, are considered [1]. Standard shoulder radiography usually includes anteroposterior (AP) projections combined with an orthogonal lateral view, such as the scapular Y view, axillary or transthoracic lateral view, apical oblique view [2], etc., to better evaluate the spatial relationship between the humeral head and glenoid cavity, while only one radiographic projection may be inadequate for definite diagnosis of an injured shoulder. Among these projections, the transthoracic lateral view provides a supplementary lateral assessment of the proximal humerus for evaluating proximal humeral fractures, fracture displacement or angulation, and glenohumeral dislocation or fracture-dislocation, especially when conventional axillary positioning is difficult to obtain [3].

Compared with the shoulder AP view, the transthoracic lateral view is more challenging because the proximal humerus is often obscured by the patient’s interposed thorax [4] and the patient’s body habitus. Along this projection, the X-ray beam must pass through both hemithoraces, causing an overlap of ribs, lung tissue, cardiac structures, and mediastinum, which would lead to image blurring and motion artifacts and compromise the image quality, thus affecting the visibility of lesion details. When complex fractures or inconclusive radiographic findings are present [5], other radiographic views or cross-sectional imaging would be combined for further diagnostic purposes. Moreover, body mass index (BMI) is an important patient-related factor influencing the image quality of shoulder radiography. The higher the BMI values, the greater the thoracic tissue thickness, leading to greater X-ray attenuation and scatter [6], which reduce detector exposure and degrade image contrast. To ensure adequate image quality, higher exposure parameters, such as increased tube current–time product (mAs) [7] and/or tube voltage, are needed. However, prolonged exposure time would increase the risk of motion artifacts and image blurring [8]. This issue would be particularly obvious for the transthoracic lateral shoulder view, which requires the X-ray beam to traverse the left-to-right thorax and is currently commonly acquired during the free-breathing mode. Respiratory motion in radiological examinations would further aggravate motion artifacts, reduce the visualization of proximal humeral and glenohumeral details, degrade imaging quality, reduce tissue contrast, and potentially interfere with lesion visualization and diagnostic confidence [9], which is particularly prominent in overweight and obese patients.

As a simple breathing control strategy, breath-holding is therefore widely used in various examinations, such as chest radiography, thoracic and abdominal CT and MR imaging, to minimize respiratory motion and improve imaging clarity and lesion detection [9,10,11,12,13]. In musculoskeletal imaging, breathing control is also applied in examinations of structures affected by respiratory movement, such as the sternum and ribs, in order to effectively reduce respiratory motion artifacts and improve lesion conspicuity [14,15,16,17]. In the shoulder transthoracic lateral view, slow breathing has been mentioned for better visualization of the proximal humerus by blurring out the overlying ribs and thoracic structures [15,18]. However, to our knowledge, no previous studies have systematically investigated the use of DIBH in transthoracic lateral shoulder radiography and its clinical impact on image quality and lesion conspicuity in patients with different BMI levels.

Therefore, there were two main objectives of this study. First, to compare the image quality of DIBH transthoracic lateral shoulder radiography with that obtained using the FB method in patients with different BMI levels when interpreted in conjunction with the corresponding AP radiograph (FB was selected as the comparator because it represents the routine respiratory condition used for transthoracic lateral shoulder radiography at our institution), and second, to clarify the capabilities of the DIBH strategy for shoulder abnormality depiction.

## 2. Materials and Methods

### 2.1. Study Population

This study was conducted in accordance with the Declaration of Helsinki and its subsequent amendments. The current prospective study was approved by the local medical ethics committee. All enrolled participants agreed to the additional DIBH method acquisition of transthoracic lateral shoulder X-ray imaging, and written informed consent was obtained.

From January 2026 to June 2026, consecutive patients with traumatic shoulder injuries and/or dislocations, undergoing postoperative follow-up, and symptomatic degenerative shoulder osseous lesions who clinically required anteroposterior (AP) and transthoracic lateral shoulder radiography were screened for eligibility. Patients who were willing to participate in this study were enrolled prospectively. Exclusion criteria: patients who were unable to stand on their feet; patients who could not perform the breath-hold maneuvers due to altered mental status, dyspnea, or other conditions; those under the age of 18; and those who declined the additional DIBH transthoracic lateral shoulder radiography, as only shoulder AP radiography was clinically required. Additionally, as this was a prospective clinical study with enrollment conducted over a predefined study period, the sample size was determined by the number of consecutive eligible patients meeting the prespecified inclusion and exclusion criteria during that period. No formal a priori sample-size calculation was performed.

### 2.2. Shoulder Radiography Protocol

A WD-CBCT600a digital X-ray radiography and fluoroscopy system (WANDONG Medical Technology Co., Ltd., Beijing, China) was used for X-ray image acquisition. The detailed imaging parameters were set according to different BMI levels of patients, taking into account the image quality and radiation protection principles, as shown in Table 1.

All participants underwent standardized shoulder radiography at a fixed source-to-image distance (SID) of 100 cm, including routine anteroposterior (AP) and standardized transthoracic lateral projections. For the AP projection, participants were positioned upright with the affected shoulder closely positioned toward the image detector. The central X-ray beam was directed perpendicular to the detector and centered inferior to the coracoid process, without tube angulation. The field of view (FOV) was adjusted to include the entire shoulder girdle, proximal humerus, glenohumeral joint, acromion, and surrounding soft tissues.

For transthoracic lateral projection, participants maintained the same upright posture, with the affected shoulder placed against the image detector. The central X-ray beam was directed horizontally through the contralateral thorax and centered on the surgical neck of the affected humerus. The FOV was adjusted to ensure complete visualization of the proximal humerus and glenohumeral joint. Before image acquisition, all participants underwent approximately five practice sessions of standardized breathing training to ensure adequate respiratory coordination. Each participant subsequently underwent two transthoracic lateral radiographic examinations in a randomized order, with an interval of 3–5 min between acquisitions to minimize potential carryover effects related to respiratory motion artifacts. For the two lateral acquisitions of each patient, identical exposure parameters (same tube voltage, tube current, and exposure time) and identical image post-processing were used, and the patient was not exposed to additional radiation beyond the two lateral views. Specifically, one image was acquired during routine FB, with participants instructed to maintain quiet and natural respiration and a stable body posture throughout the exposure. The other image was obtained using a DIBH method, in which participants were instructed to inhale as deeply as possible and then maintain a stable breath-hold for approximately 3 s before and during the exposure. Radiographic exposure was only initiated after confirmation of stable body positioning and required respiratory status, thereby ensuring consistency and image quality across participants.

### 2.3. Image Quality Evaluation

Two experienced musculoskeletal radiologists, with 15 (Q.G.C.) and 25 years (Z.Q.L.) of working experience, respectively, independently evaluated all images. The readers were unaware of the acquisition labels and were not provided with information regarding the respiratory condition during image evaluation. All images were fully anonymized and stripped of patient-identifying information and acquisition labels that might have indicated the respiratory condition during the reading sessions. The image sets were assigned randomized identifiers and presented to the readers in a randomized order. The readers were not informed whether the lateral radiograph had been acquired using the FB or DIBH technique. Because transthoracic lateral projection cannot be used independently for lesion visualization or comprehensive assessment in clinical practice, the AP + FB and AP + DIBH image sets were evaluated separately. The AP component was identical between the two image sets, while the lateral projection was the only variable. This paired design was intended to isolate the incremental effect of the lateral imaging technique under conditions that reflect routine clinical image interpretation. Therefore, the AP image served as a constant component view for anatomical and abnormal assessments, and the subjective scoring was only held for the lateral view. To reduce recall bias, image sets were evaluated in two separate reading sessions conducted one month apart, and within each session the order of the patients was randomized.

- Qualitative assessment

The image quality was qualitatively scored according to a 4-point scale (1, poor; 4, excellent) regarding visualization of anatomical structures and capability for lesion conspicuity under the two respiratory protocols (Table 2). Given the inclusion criteria, the abnormality assessment mainly focused on fractures, dislocations, and postoperative implants in the current study. Lesion conspicuity scoring was only performed in patients with focal structural bony abnormalities visible on the radiographs (fresh or obvious fracture lines, traumatic dislocations, metallic implants, or focal bony defects), whereas patients with only diffuse soft-tissue changes or healed lesions without a focal bony abnormality were excluded from the lesion evaluation, yielding 50 evaluable lesion-positive cases among the 128 examinations. To eliminate memory bias, the FB and DIBH image sets were evaluated in two separate reading sessions conducted one month apart. During each session, radiologists reviewed the AP image together with the lateral image acquired using only one of the respiratory methods, FB or DIBH. The order of the FB and DIBH methods was randomly assigned rather than fixed. All images were viewed and analyzed on the workstation of the WD-CBCT600a system (WANDONG Medical Technology Co., Ltd., Beijing, China) under standardized viewing conditions. The two radiologists were allowed to use the zoom-in and zoom-out functions to observe image details for diagnostic purposes.

- Quantitative Assessment

**Figure 1:**
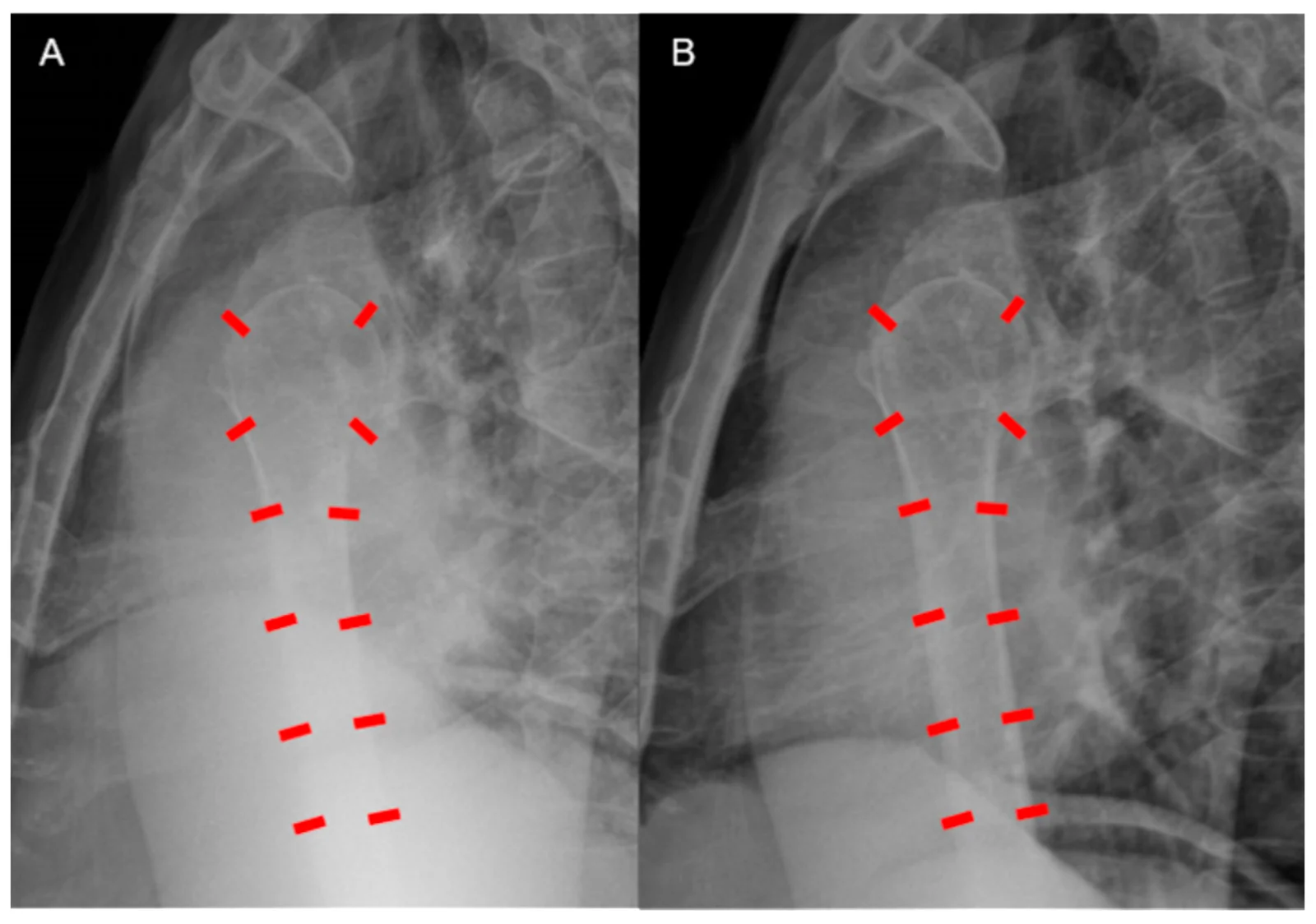
The placement of short lines for measuring edge sharpness of the humerus and adjacent tissues using ImageJ software on free-breathing (**A**) and deep-inspiration breath-hold (**B**) transthoracic lateral shoulder radiographs.

For quantitative analysis, ImageJ software 1.53t (https://imagej.net/software/imagej/ (accessed on 1 May 2026)) was used to measure the edge sharpness [19] of the humerus and adjacent tissues in FB and DIBH images. A single radiology resident (Y.H.S.) who was blinded to patient identity and to the respiratory protocol placed the measurement lines at 12 standardized anatomical locations (e.g., humeral head cortex, greater tuberosity, surgical neck, and the posterior humeral shaft boundary) with the same distance between adjacent lines (illustrated as Figure 1); for each patient, the corresponding locations on the FB and DIBH images were matched using identical anatomical landmarks, and the per-line values were treated as internal replicates rather than independent observations. A 0.3 cm wide line was manually drawn, extending from the humeral surface to the adjacent tissues. The software automatically generated a grayscale value distribution curve (X-axis: line segment length, Y-axis: signal intensity), and the local maximum (Max) and minimum (Min) values at the boundary in the Y-axis direction were determined. Then, the distance (d) between [0.8 × (Max − Min) + Min] and [0.2 × (Max − Min) + Min] in the X-axis direction was measured, and the edge sharpness was calculated as 1/d mm. The edge sharpness of each image was the average of the 12 line measurements. All measurements were performed on the same DICOM images with identical window/level settings. Identical post-processing algorithms (default vendor processing) were applied to all images; no manual adjustments were made before ES analysis. Edge sharpness is a core quantitative index reflecting the detail display ability of X-ray images; the larger the value, the clearer the structural edges and the stronger the detail recognition capability.

- Visualization of shoulder abnormalities

One month after the qualitative and quantitative evaluations mentioned above, the two radiologists reviewed the shoulder abnormalities together to reach a consensus as a final diagnostic result. A third radiologist with 30 years of experience (Z.Q.L.) acted as an arbiter if a consensus was not reached.

- CT-based subgroup analysis

Among patients who underwent CT, an additional subgroup analysis was performed to compare the radiographic assessment with CT-confirmed findings. CT was used to confirm the presence and type of shoulder abnormalities in this subgroup. The CT-confirmed abnormalities were categorized as isolated humeral fracture, fracture-dislocation, or degenerative disease. The diagnostic interpretation of the AP + FB and AP + DIBH image sets was compared with the corresponding CT findings. This analysis was intended to assess concordance with CT-confirmed diagnoses rather than formal diagnostic performance, because CT was not available for the entire study population and no uniform independent reference standard was available for all patients.

### 2.4. Statistical Analysis

Image quality scores were expressed as median and interquartile range (IQR). The primary outcome was the subjective anatomical depiction score and the quantitative edge sharpness of the transthoracic lateral projection, compared between DIBH and FB within the same patient. The Wilcoxon signed-rank test was used to compare ordinal subjective image quality scores between routine FB and DIBH methods. Inter-observer agreement between the two radiologists for subjective scores of the two methods was analyzed using linear weighted kappa statistic (weights 1 − |i − j|/(k − 1); <0.40, poor; 0.40–0.59, moderate; 0.60–0.75, good; >0.75, excellent), with confidence intervals estimated by non-parametric bootstrap and truncated to the valid [−1, 1] range. McNemar’s test was applied to compare the proportion of acceptable images (score ≥ 3) for anatomical structure and lesion visualization between FB and DIBH methods, and the discordant-pair counts are reported. The Shapiro–Wilk test was first utilized to verify the normality assumption of the ES differences; subsequently, the paired t-test was adopted to compare ES values between the two methods, and a post hoc power analysis was performed based on the observed paired effect size. The Kruskal–Wallis H test combined with Bonferroni post hoc correction was performed to conduct pairwise comparisons of visualization scores across the three BMI subgroups, and one-way ANOVA was applied to compare the ES values across BMI subgroups. To test whether the benefit of DIBH varied with BMI grouping, cumulative-link mixed-effects models (ordinal logistic mixed models) with a random intercept per subject were fitted for the subjective scores, with fixed effects of respiratory mode, BMI group, and their interaction. The linear mixed-effects model with a random intercept per subject was the primary inferential model for repeated ES measurements across the two methods (FB and DIBH) and the three BMI subgroups. The model was fitted by restricted maximum likelihood, with fixed effects of BMI group, scan mode, and their interaction, thereby directly testing the BMI × mode interaction while accounting for the clustering of repeated measurements within each subject. For the CT subgroup, concordance between the radiographic diagnosis based on the AP + FB or AP + DIBH image set and the corresponding CT-confirmed diagnosis was summarized descriptively as the number and percentage of concordant cases. All the statistical analyses were performed using SPSS software (IBM SPSS 22, IBM Corp., Armonk, NY, USA). *P*-values of 0.05 or less were considered to be statistically significant.

## 3. Results

**Figure 2:**
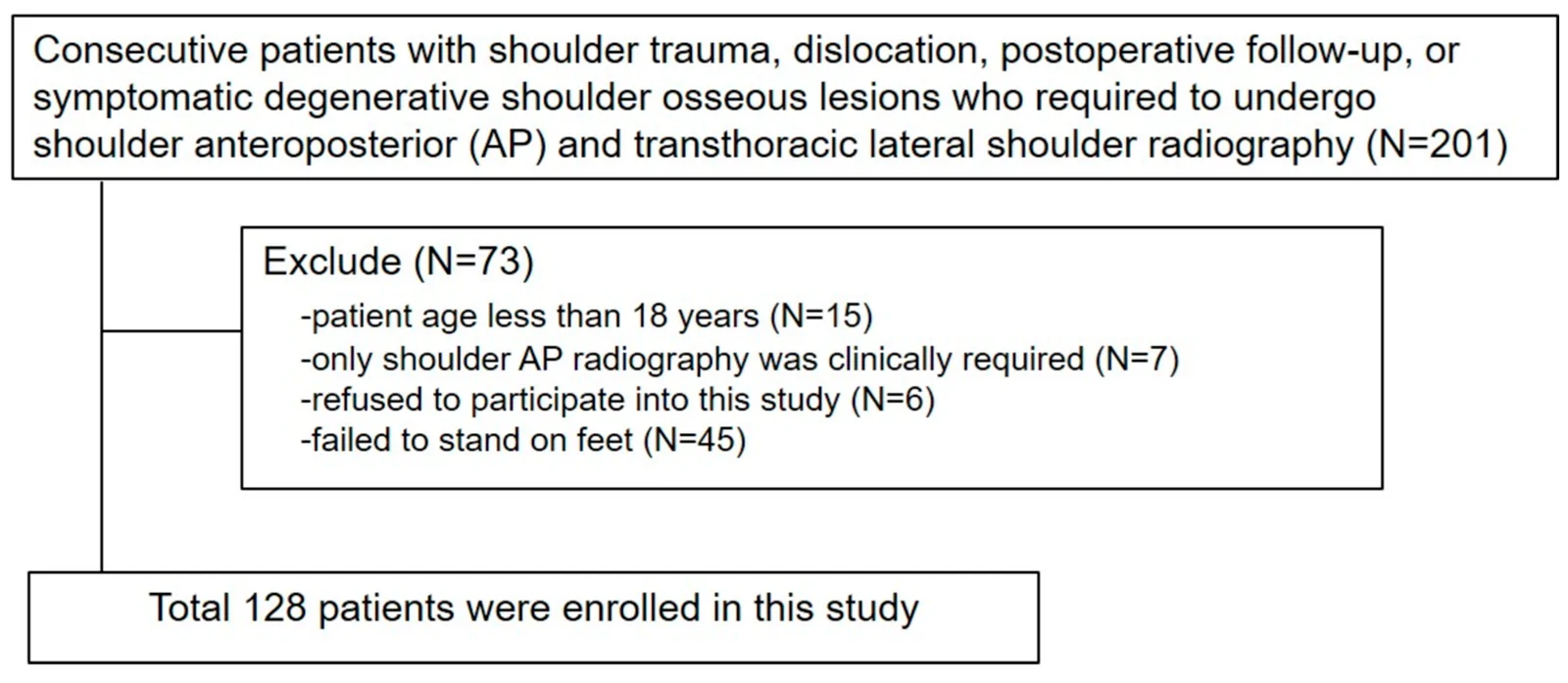
Patient selection flowchart.

A total of 128 patients (mean age, 56.1 ± 12.3 years; range 32–78 years; 68 males, 60 females) completed the required radiography with FB and DIBH transthoracic lateral shoulder radiography (Figure 2, Table 3). All the enrolled patients completed the required shoulder radiographs successfully without any discomfort. The cohort identified by shoulder radiographs included patients with isolated humeral fracture (*n* = 38), fracture-dislocation (*n* = 14), postoperative follow-up examinations (*n* = 40), and degenerative diseases (*n* = 36). Based on BMI, 70 patients had a BMI < 24 kg/m^2^, 46 had a BMI of 24–28 kg/m^2^, and 12 had a BMI ≥ 28 kg/m^2^. The detailed distribution of the 50 lesion-evaluable cases has been summarized in Table 4.

### 3.1. Subjective Image Quality Assessment

**Figure 3:**
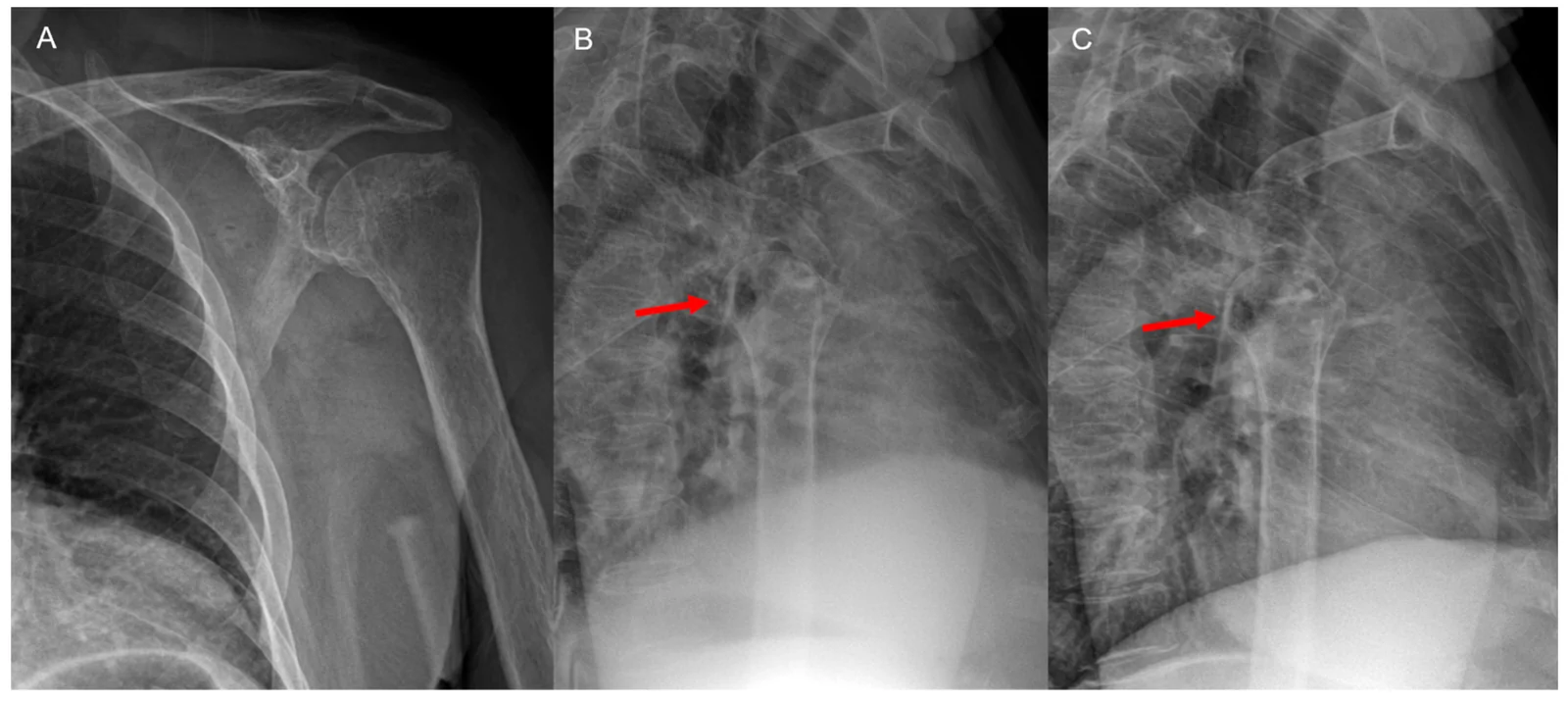
Shoulder X-ray radiographs of a 65-year-old male patient with degenerative shoulder disease. (**A**) Anteroposterior shoulder X-ray image showed focal cortical hyperostosis. The anatomical marginal delineation of the humerus was significantly inferior, as shown on routine free-breathing (**B**), when compared with a deep-inspiration breath-hold transthoracic lateral shoulder radiograph (**C**) (arrows).

**Figure 4:**
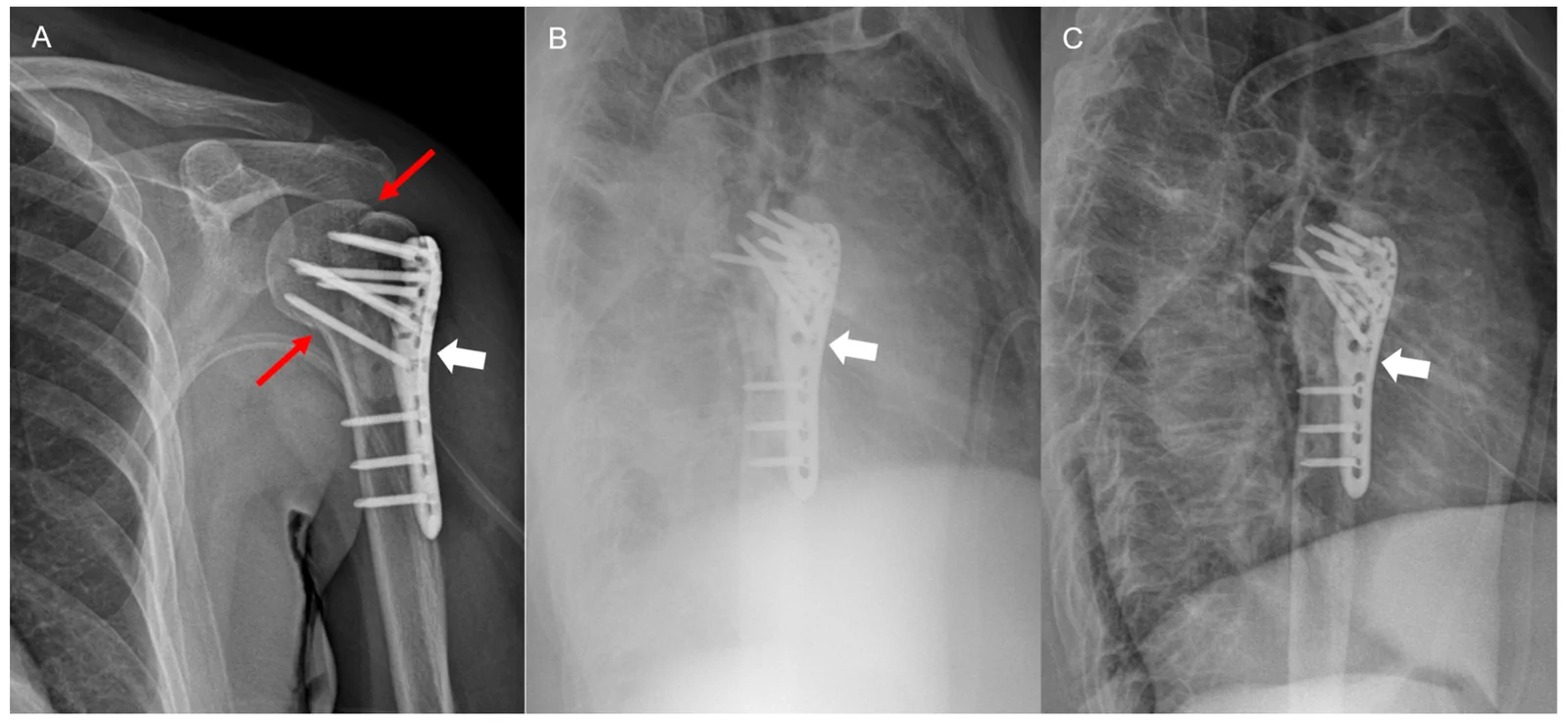
Shoulder radiographs of a 46-year-old male patient after humeral head fracture fixation. (**A**) Anteroposterior shoulder projection demonstrated the humeral head fracture (red arrow) and the metallic implant (white arrow). Compared with the free-breathing image (**B**), the deep-inspiration breath-hold image (**C**) showed greater lung expansion and a lower diaphragmatic position, resulting in a more radiolucent thoracic background and reduced superimposition of thoracic soft tissues over the proximal humerus. The cortical margins of the proximal humerus and the fixation metallic implant were more clearly delineated (white arrows), with improved image contrast and less motion-related blurring.

**Figure 5:**
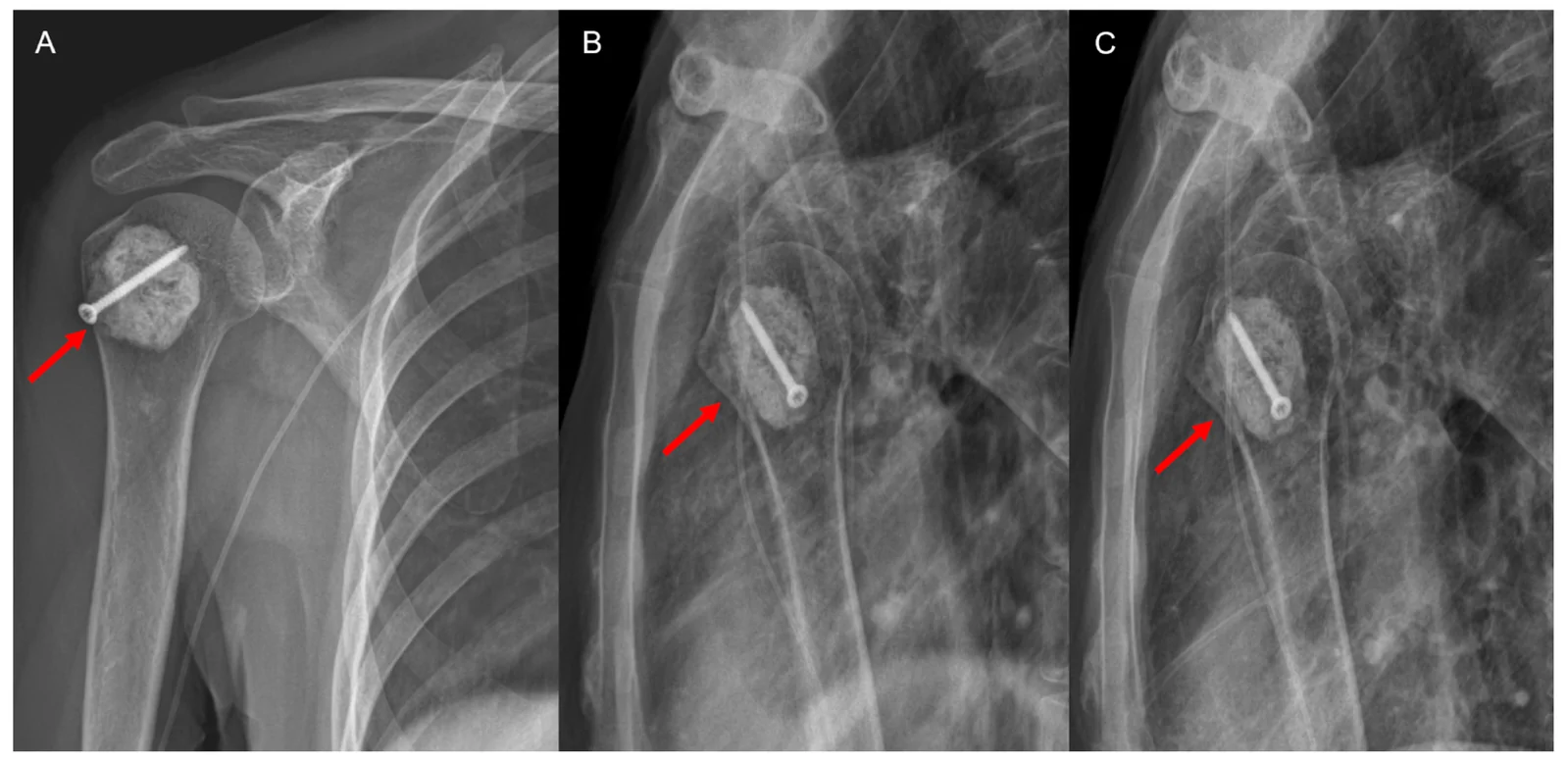
Shoulder radiographs of a 57-year-old male patient with a humeral head tumor. (**A**) The anteroposterior shoulder radiograph showed the postoperative region of the proximal humerus (red arrow). Compared with the transthoracic lateral radiograph acquired by the free-breathing method (**B**), the corresponding lateral radiograph acquired using the deep-inspiration breath-hold technique (**C**) showed increased lung aeration and reduced soft-tissue superimposition, with clearer delineation of the proximal humeral cortex and implanted screw (red arrow).

**Figure 6:**
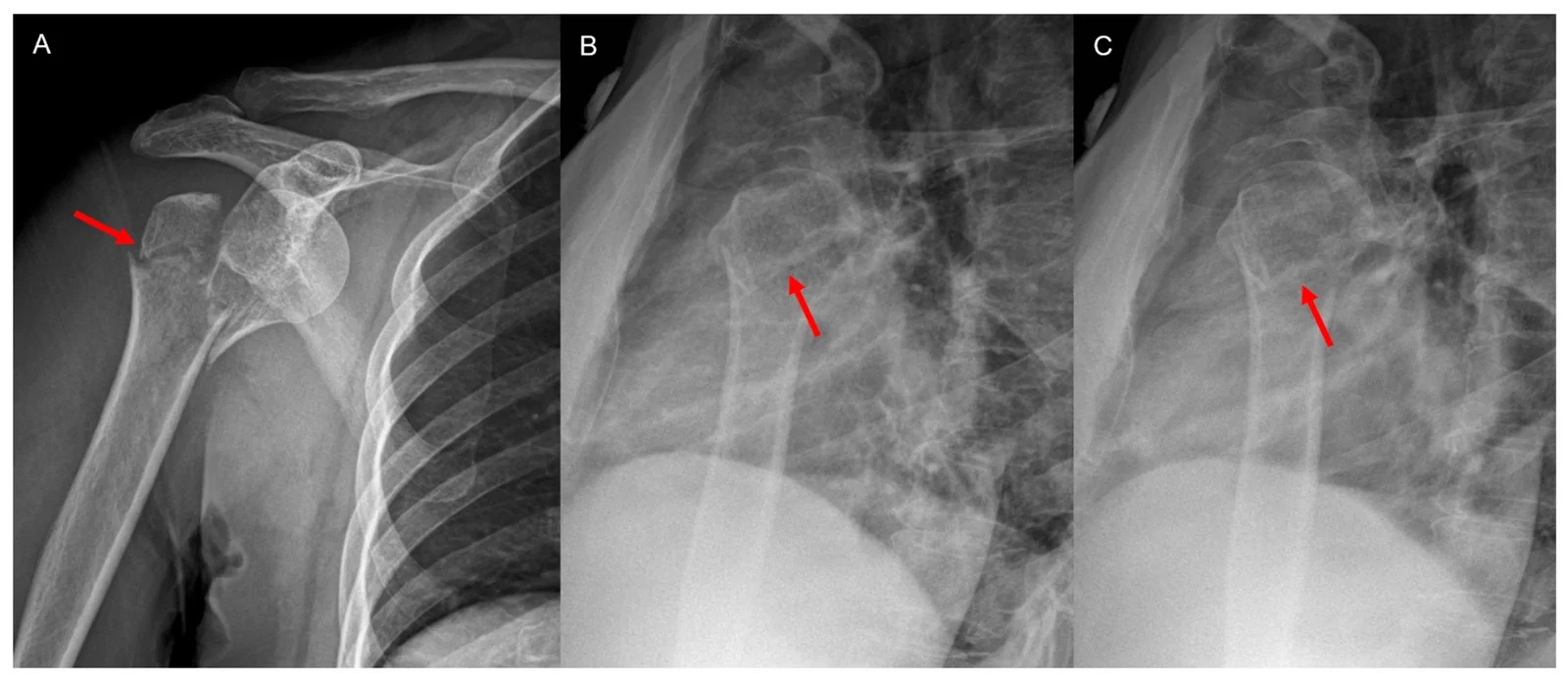
Shoulder radiographs of a 35-year-old male patient with preoperative humeral head fracture-dislocation. (**A**) Anteroposterior shoulder radiograph showed the humeral head fracture-dislocation (red arrow). (**B**) Transthoracic lateral radiograph obtained using the routine free-breathing method, showing relatively poor conspicuity of the fracture because of the thoracic soft-tissue superimposition and respiratory motion blurring, which could easily be mistaken for the margin of a rib. (**C**) Corresponding lateral radiograph acquired using the deep-inspiration breath-hold method, demonstrating improved image contrast and sharper delineation of the fracture and adjacent cortical margins (red arrow) when compared with those in (**B**).

For anatomical structural depiction, the scores of DIBH images achieved superior performance compared with those of FB images when interpreted in conjunction with the corresponding AP radiograph. Reader 1 rated the FB images as 3.0 (IQR, 3.0–3.0) and the DIBH images as 3.5 (IQR, 3.0–4.0) (z = −8.014, *p* < 0.001). Reader 2 assigned 3.0 (IQR, 3.0–3.0) for the FB images and 3.0 (IQR, 3.0–4.0) for the DIBH images (z = −7.616, *p* < 0.001) (Figure 3, Figure 4, Figure 5 and Figure 6). Anatomical depiction scores improved in 70 (54.7%) and 58 (45.3%) of 128 patients, remained unchanged in 56 (43.8%) and 70 (54.7%), and worsened in 2 (1.6%) and 0 (0.0%) for readers 1 and 2, respectively.

For lesion depiction, the scores of DIBH images were significantly higher than those of FB images for reader 1 (*p* = 0.005) and reader 2 (*p* = 0.046). Reader 1 rated the FB images as 3.0 (IQR, 3.0–4.0) and the DIBH images as 3.0 (IQR, 3.0–4.0) (z = −2.828, *p* = 0.005). Reader 2 assigned 3.0 (IQR, 2.0–4.0) for the FB images and 3.0 (IQR, 3.0–4.0) for the DIBH images (z = −2.000, *p* = 0.046) (Figure 5 and Figure 6). Lesion conspicuity scores improved in 8 (16.0%) and 4 (8.0%) of 50 patients, remained unchanged in 42 (84.0%) and 46 (92.0%), and worsened in 0 (0.0%) for readers 1 and 2, respectively.

Image acceptability analysis (proportion of images with score ≥ 3) (Table 5) revealed that the proportions of acceptable images in the DIBH method were significantly superior to those in the FB method for both readers for anatomical depiction (Reader 1: 126/128 vs. 108/128, *p* < 0.001; Reader 2: 126/128 vs. 106/128, *p* < 0.001). For lesion depiction, the proportions were higher in the DIBH method for both readers, but the difference was statistically significant only for Reader 1 (Reader 1: 44/50 vs. 38/50, *p* = 0.031; Reader 2: 40/50 vs. 36/50, *p* = 0.125).

Moderate-to-excellent inter-reader consistency was observed. For anatomical depiction scores, moderate inter-reader agreement was observed for the FB method (weighted κ = 0.546, 95% CI 0.39–0.68, *p* < 0.001), while good agreement was achieved for the DIBH method (weighted κ = 0.617, 95% CI 0.48–0.74, *p* < 0.001). For lesion visualization scores, excellent inter-reader agreement was demonstrated for both the FB method (weighted κ = 0.788, 95% CI 0.64–0.90, *p* < 0.001) and the DIBH method (weighted κ = 0.857, 95% CI 0.73–0.95, *p* < 0.001). Weighted kappa was calculated with linear weights, and confidence intervals were estimated by non-parametric bootstrap and truncated to the valid [−1, 1] range.

### 3.2. Quantitative Evaluation

**Figure 7:**
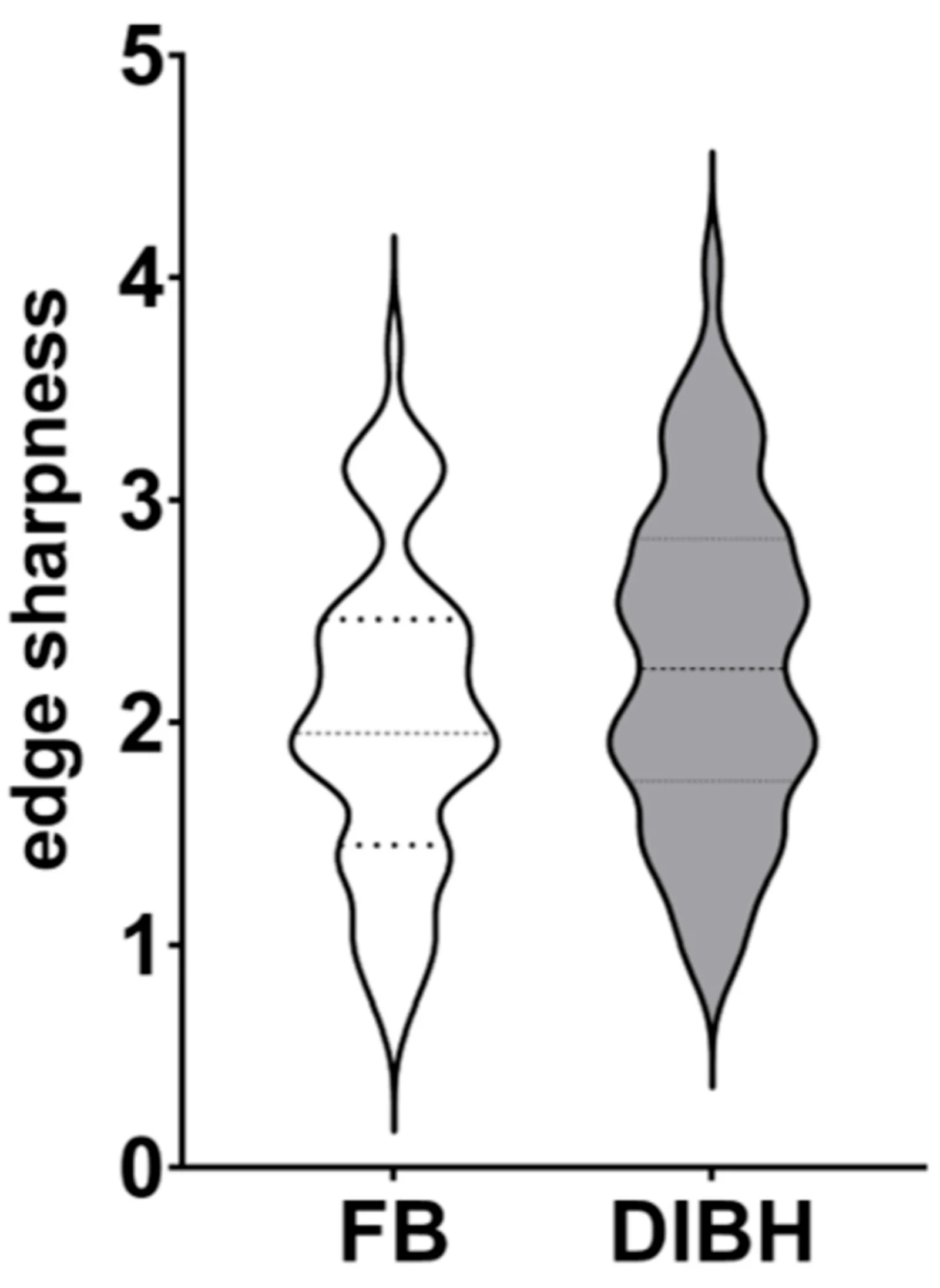
Violin plot of edge sharpness values of transthoracic lateral shoulder radiography applying free-breathing (FB) and deep-inspiration breath-hold (DIBH) strategies.

The ES of the DIBH method was statistically higher than that of the FB method (2.27 ± 0.73 vs. 2.02 ± 0.70; paired t = 4.80, *p* < 0.001; mean difference 0.24, 95% CI 0.14–0.34; Cohen’s dz = 0.42; Figure 7).

### 3.3. Analyses Across BMI Subgroups

Anatomical depiction scores for FB images differed significantly across BMI subgroups for both readers (reader 1: H = 6.374, *p* = 0.041; reader 2: H = 7.321, *p* = 0.026). For DIBH images, no statistically significant differences across BMI subgroups were observed for anatomical depiction scores (reader 1: H = 4.303, *p* = 0.116; reader 2: H = 4.978, *p* = 0.083) or for lesion depiction scores (reader 1: H = 4.926, *p* = 0.085; reader 2: H = 5.392, *p* = 0.067). For FB images, lesion depiction scores differed significantly across BMI subgroups only for reader 2 (H = 6.076, *p* = 0.048), but not for reader 1 (H = 5.330, *p* = 0.070) (Table 6). To evaluate whether the benefit of DIBH varied with BMI, cumulative-link mixed-effects models (ordinal logistic models with subject-level random intercepts) were fitted for the subjective scores, including respiratory mode, BMI grouping, and their interaction. The mode × BMI interaction was not significant for anatomical depiction scores (reader 1: LR χ^2^ = 0.971, df = 2, *p* = 0.616; reader 2: LR χ^2^ = 0.366, df = 2, *p* = 0.833) or for lesion depiction scores (reader 1: LR χ^2^ = 0.177, *p* = 0.915; reader 2: LR χ^2^ = 5.394, *p* = 0.067). One-way ANOVA revealed no statistically significant difference in ES values among the three BMI subgroups for FB (F = 1.977, *p* = 0.143) or the DIBH method (F = 2.020, *p* = 0.137). The linear mixed-effects model revealed no significant interaction between BMI grouping and respiratory mode regarding ES measurement (F = 0.061, df = 2, 125, *p* = 0.941). The main effect of respiratory mode was significant (DIBH vs. FB mean difference 0.227, SE 0.066; F = 11.791, *p* = 0.001; ICC = 0.67), whereas the main effect of BMI grouping was not significant (F = 2.381, *p* = 0.097).

### 3.4. CT-Confirmed Findings

**Figure 8:**
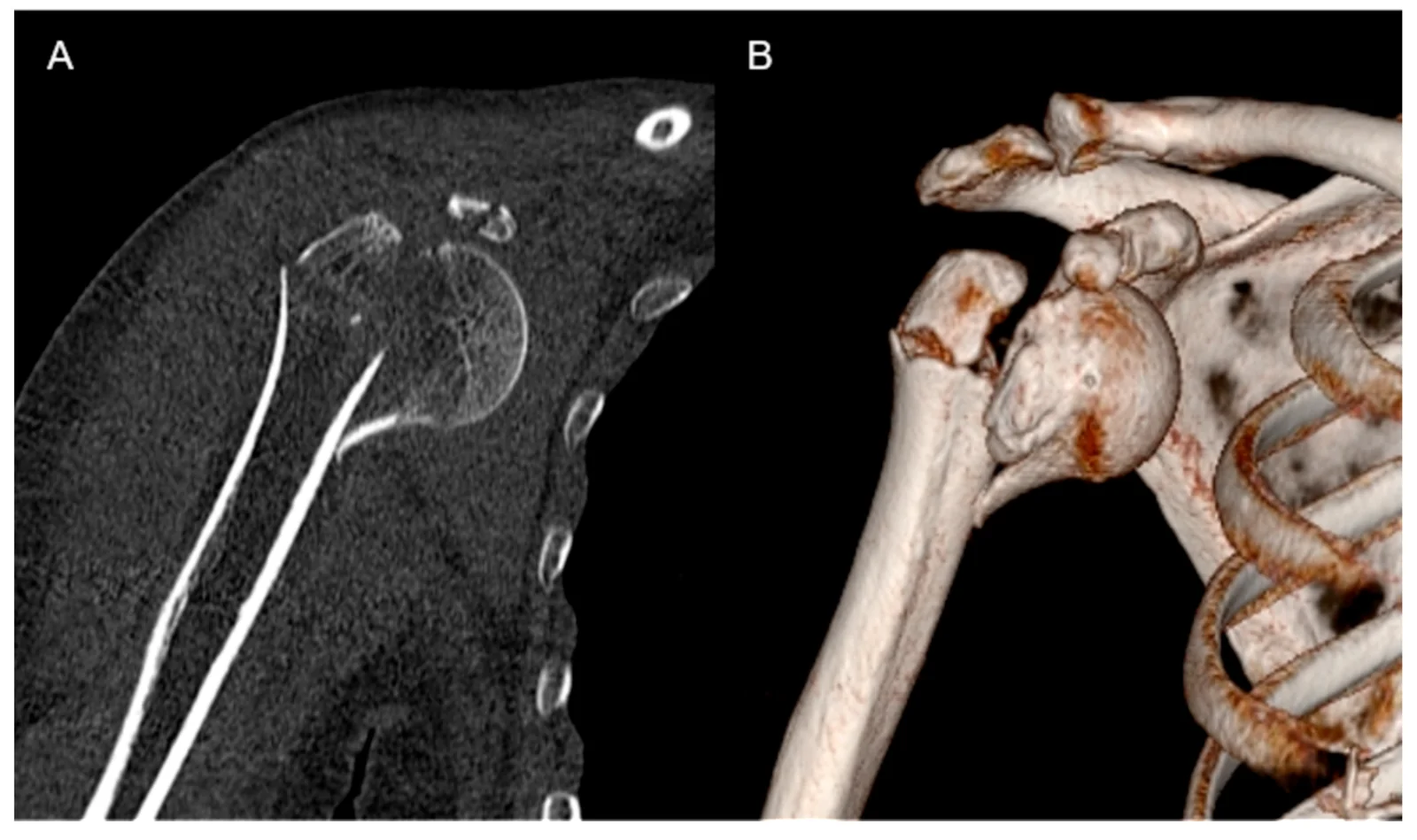
CT images of the right shoulder in the same patient as shown in Figure 6. Coronal multiplanar reconstruction (MPR) (**A**) and volume-rendered (VR) (**B**) images clearly demonstrated a comminuted fracture of the right humeral head and neck with dislocation of the right shoulder joint. The findings on the preceding shoulder radiographs were concordant with the CT findings.

Among all 128 enrolled patients, 72 subjects received a shoulder CT scan for further verification after initial X-ray examination. CT-confirmed abnormalities included isolated humeral fractures in 38 patients (52.8%), fracture-dislocations in 14 patients (19.4%) (Figure 8), and degenerative diseases in 20 patients (27.8%). The diagnosis based on both the AP + FB and AP + DIBH image sets was totally concordant with the corresponding CT findings in all 72 patients (72/72, 100%). Thus, no difference in diagnostic concordance with CT was observed between the two image sets in this subgroup.

## 4. Discussion

In the current study, we compared the subjective image quality and the quantitative edge sharpness of the deep-inspiration breath-hold strategy in transthoracic lateral shoulder radiography with those of the routine free-breathing method. Both qualitative and quantitative assessments supported that the breath-hold strategy improved image quality of the transthoracic lateral projection when interpreted in conjunction with the corresponding AP radiograph in clinical practice.

The transthoracic lateral radiography of the shoulder joint is technically challenging, primarily because of its complex anatomical configuration. The humeral head, glenoid fossa, acromion, and surrounding osseous structures overlap with each other, while also being superimposed over intrathoracic tissues, including the myocardium, lung parenchyma, mediastinum, ribs, and adjacent soft tissues. The conventional lateral shoulder radiographic protocols are generally performed with patients breathing freely; however, respiratory motion can induce subtle displacement of the shoulder girdle during image acquisition, resulting in motion blur and reduced image sharpness. This issue may be particularly obvious in overweight and obese patients, in which longer exposure time is usually set in the imaging protocol, thereby increasing the respiratory artifacts. Consequently, accurate visualization of anatomical landmarks and pathological changes may be compromised by both anatomical overlap and motion-related image degradation, particularly in musculoskeletal imaging where detection of fine cortical abnormalities is required [20,21].

Respiratory-related motion during image acquisition can produce edge blurring, reduce spatial resolution, and impair the visibility of small anatomical structures or pathological changes. Thus, respiratory motion is routinely controlled in thoracic and abdominal radiological examinations through breath-hold instructions to minimize motion artifacts and improve image quality. Although full inspiration has been mentioned for transthoracic lateral shoulder projection [18], the influence of respiratory control on shoulder radiography has received relatively limited attention. Through comparison of transthoracic lateral shoulder radiography applying routine free-breathing and DIBH, our results indicate that DIBH represented an effective and simple approach in clinical practice, which is consistent with the general concept of radiographic optimization to achieve superior image quality while maintaining a simple and practical acquisition procedure [22].

The improvement of image quality observed in the current study in the deep inspiration method when interpreted in conjunction with the corresponding AP radiograph likely contributes to both increased aeration of the thorax and respiratory suspension. The volume of intrapulmonary air increases after DIBH, whereas the relative density of the thorax is decreased when compared with the condition with free breathing, allowing a larger proportion of the primary X-ray beam to reach the detector after passing through the aerated thorax. Therefore, the margins of the humeral head and adjacent osseous structures have been more clearly distinguished from the superimposed myocardium, mediastinum, ribs, lung tissues, and soft tissues. Moreover, with the help of the breath-hold method, the motion-related artifacts and image blurring have been reduced. Although the intrinsic X-ray beam energy has not been changed, the lower attenuation associated with increased pulmonary aeration improves the X-ray transmission through the overlapping thorax, thus providing better image quality and cortical edge sharpness, which help to depict abnormalities in musculoskeletal radiography, such as fractures, degenerative alterations, and structural deformities, and thereby increasing the diagnostic confidence during the image interpretation.

In our study, no significant interaction effect was observed between respiratory status and the three BMI groups, and the absence of a significant interaction should not be interpreted as evidence of equal benefit across BMI groups given the limited number of patients in the highest BMI group (*n* = 12). One possible explanation is that, under identical X-ray exposure conditions, patients with a higher BMI would be expected to exhibit poorer image quality and reduced tissue contrast than those with a lower BMI. In such circumstances, the improvement in image quality during inspiration might be more pronounced in patients with a higher BMI. However, in routine clinical practice, exposure parameters are typically adjusted according to the patient’s BMI under automatic exposure control, which may compensate for BMI-related differences in image quality and thereby explain the absence of a significant interaction between respiratory status and BMI group.

The breath-hold strategy used in this study has offered clearer anatomical outlines and better improvement in lesion conspicuity, which can be readily incorporated into routine transthoracic lateral shoulder radiography. Unlike modifications involving increased radiation exposure or additional equipment, respiratory control does not require changes in imaging parameters and represents a zero-cost optimization strategy; importantly, the FB and DIBH lateral views were obtained with identical exposure parameters, so the DIBH acquisition itself did not increase the radiation dose per image. This is particularly practical for shoulder radiography, which remains the initial imaging modality for shoulder disorders due to its accessibility, rapid acquisition, and relatively low radiation burden compared with advanced imaging techniques such as CT [23]. Based on the findings of the current study, we recommend the breath-hold method as a simple, zero-cost, and clinically practical strategy for improving the image quality of transthoracic lateral shoulder radiography.

There are several limitations of this study. First, this single-center study enrolled a limited sample size, particularly for the higher-BMI subgroup (only 12 of 128, 9.4%), mainly due to the public’s growing emphasis on health and weight management; the study was not prospectively powered for the BMI-subgroup comparisons, and a post hoc power analysis based on the observed ES effect size indicated high statistical power for the ES comparison. Large-scale, multicenter studies are required in the future. Second, this study assessed the subjective image quality of FB and DIBH lateral radiographs with respect to the visualization of anatomical structures and abnormalities when interpreted in conjunction with the corresponding AP radiograph. This approach was intentionally chosen to reflect routine clinical practice, in which AP and lateral radiographs are typically interpreted together. Therefore, the study was designed to evaluate the incremental effect of the FB or DIBH lateral imaging technique within a clinically relevant image set, rather than to compare lateral radiographs acquired under different respiratory conditions in isolation. Moreover, the edge sharpness assessment was only performed on lateral images without the combination of AP views, which would provide additional evidence to support our conclusion. Third, a limited disease spectrum was included in this study, and more disease types will be considered in the next step to enable disease-specific statistical analyses. Fourth, patients were instructed to take a deep breath and hold it through verbal training and commands. However, due to differences in disease conditions and patient cooperation, the effectiveness of deep inspiration varied among patients, and the current study did not include a quantitative evaluation of this factor; we are unable to make a judgment on whether the breath-holding was poor or failed, which may have introduced some bias to the final results. In future studies, we will consider methods, such as a visualized respiration training device [16] or the help of a spirometer and visual feedback [24], to quantify respiratory movements. Fifth, the incremental clinical value of the DIBH strategy for long-term follow-up patients and for a large population of patients with shoulder disorders still requires further investigation and validation. Sixth, although CT was available for some of the enrolled patients for verification and was used to confirm shoulder abnormalities, not all the patients underwent CT, and no uniform independent reference standard was available for the entire cohort. Therefore, the present study primarily demonstrates improvement in image quality and lesion conspicuity and does not establish whether the DIBH method improves diagnostic accuracy, sensitivity, or specificity, which still needs more validation in further research. Seventh, another limitation is that complete blinding of the readers to the respiratory condition was not possible. Although the images were anonymized, randomized, and stripped of acquisition information indicating the imaging protocol, intrinsic radiographic features, such as lung expansion and diaphragmatic position, may have allowed readers to infer whether the image was acquired during FB or DIBH. This may have introduced a degree of observer bias in the subjective image-quality assessment in this study. Eighth, although slow breathing has been mentioned to blur superimposed thoracic structures during transthoracic lateral shoulder radiography, its implementation can be difficult to standardize because respiratory depth and frequency vary among patients and depend on patient cooperation. FB was selected as the comparator because it is the routine technique used at our institution. Therefore, future studies directly comparing FB, slow breathing, and DIBH are still warranted. Ninth, the sample size in the current study was determined by the number of eligible patients recruited during this period, and no formal a priori sample-size calculation was performed. The post hoc calculation does not replace a respecified sample-size determination, which was also a limitation of this study. Tenth, the absence of a statistically significant interaction between BMI category and breathing condition should not be interpreted as evidence of equivalent DIBH benefit across BMI strata. This is particularly important given the small number of patients in the BMI ≥ 28 kg/m^2^ subgroup (*n* = 12), which limits the statistical power to detect effect modification by BMI. This was exploratory and still needed more validation in our next step. Eleventh, although the DIBH strategy did not require a higher radiographic exposure dose than the FB method under the same acquisition protocol, the additional radiation dose of DIBH acquisition was not clinically available on the DR system used in our center. We plan to further communicate with the manufacturer regarding the availability of those dose metrics and will investigate this issue in future studies.

## 5. Conclusions

Overall, our findings suggest that deep-inspiration breath-hold acquisition is a feasible and effective method for optimizing transthoracic lateral shoulder radiography, particularly in cooperative patients. This strategy improves image sharpness and enhances subjective image quality, including anatomical depiction and lesion conspicuity. Although the CT subgroup evaluation showed similar diagnostic concordance between the FB and DIBH approaches, the improved image quality may facilitate clinical interpretation and enhance diagnostic confidence in routine practice, without increasing radiation dose per image or examination complexity. Based on these findings, the DIBH method has been incorporated into our institutional imaging practice, particularly for patients in whom thoracic superimposition compromises image quality, including those with greater thoracic soft-tissue thickness. Conventional free-breathing imaging may remain appropriate for patients who are unable to adequately follow the breath-holding instructions. Considering its simplicity and applicability, breath-hold acquisition is recommended to be integrated into routine radiographic practice for lateral shoulder radiography.

## Figures and Tables

**Table 1:** The detailed radiographic X-ray exposure parameters of body mass index (BMI) subgroups.

BMI (kg/m^2^)	Tube Voltage (kV)	Tube Current (mA)	Exposure Time (mS)	Source-Image Distance (SID, cm)
<24	75	200	180	100
24≤ <28	85	320	160	100
≥28	90	200	250	100

**Table 2:** Criteria for qualitative image quality comparisons between FB and DIBH transthoracic lateral shoulder radiography.

**Anatomical Structure Visualization Score**
4 (Excellent) Shoulder osseous structures are clearly delineated with intact cortex and distinct trabeculae. Joint and periarticular anatomy are well-layered, without artifacts or noise, for high-resolution diagnosis.
3 (Good) Major bony and articular structures are well visualized with clear bone texture. Mild noise or artifacts are present but do not interfere with routine anatomical assessment.
2 (Fair) Anatomical structures are vaguely defined with blurred margins and poor bone detail. Obvious artifacts limit fine observation, only allowing rough localization.
1 (Poor) Severe blurring, artifacts, and noise obscure bony anatomy and tissue boundaries, precluding valid anatomical evaluation.
**Abnormality Visualization Score**
4 (Excellent) Lesion margins are sharp, with clear visualization of morphology, scope, and detailed features. Lesion manifestations are fully displayed to support accurate diagnosis and classification.
3 (Good) Lesion location and nature are identifiable with clear boundaries for definite diagnosis. Minor subtle detail defects do not affect clinical interpretation.
2 (Fair) Lesions are poorly defined with blurred edges. Morphological and scope evaluation is limited, failing to achieve definitive diagnosis.
1 (Poor) Severe image degradation and artifacts obscure lesions, resulting in undetectable abnormal findings.

FB, free-breathing; DIBH, deep-inspiration breath-hold.

**Table 3:** Baseline characteristics of the enrolled subjects.

Characteristic	All Patients	BMI < 24 kg/m^2^	BMI 24 ≤ < 28 kg/m^2^	BMI ≥ 28 kg/m^2^
Patient number, *n*	128	70	46	12
Age, years	56.1 ± 12.3	54.7 ± 11.8	57.6 ± 13.1	58.3 ± 12.7
Male/female, *n*	68/60	37/33	24/22	7/5
Disease classification, *n*				
Isolated humeral fracture	38	22	13	3
Fracture-dislocation	14	8	5	1
Postoperative follow-up	40	21	15	4
Degenerative shoulder disease	36	19	13	4

BMI, body mass index. Data are presented as mean ± SD or numbers.

**Table 4:** Distribution of the 50 lesion-evaluable cases by lesion subtype.

Lesion Subtype	*n*	Proportion of Lesion-Evaluable Cases
Isolated humeral fracture with clear fracture lines	26	52.0%
Fracture-dislocation	14	28.0%
Postoperative internal fixation hardware with visible implant–bone interfaces	6	12.0%
Focal degenerative bony lesions	4	8.0%
Total	50	100%

Lesion conspicuity scoring was only performed in patients with fresh or focal structural bony abnormalities visible on radiographs (discrete fracture lines, traumatic dislocations, metallic implants, or focal bony defects). Patients with old, healed fractures or only diffuse soft-tissue degenerative changes without focal bony lesions were excluded from the lesion evaluation.

**Table 5:** Analysis of acceptable images (score ≥ 3) for anatomical structure and lesion visualization between the FB and DIBH methods.

Reader	Evaluation Type	Total, *n*	FB, *n* (%)	DIBH, *n* (%)	*p* Value
Reader 1	Anatomical structure score	128	108 (84.4)	126 (98.4)	<0.001
	Lesion conspicuity score	50	38 (76.0)	44 (88.0)	0.031
Reader 2	Anatomical structure score	128	106 (82.8)	126 (98.4)	<0.001
	Lesion conspicuity score	50	36 (72.0)	40 (80.0)	0.125

FB, free-breathing. DIBH, deep-inspiration breath-hold.

**Table 6:** Subjective score comparisons across the three BMI groups.

	Reader 1				Reader 2			
	FB Method		DIBH Method		FB Method		DIBH Method	
	Anatomical Structure Score	Lesion Visualization Score	Anatomical Structure Score	Lesion Visualization Score	Anatomical Structure Score	Lesion Visualization Score	Anatomical Structure Score	Lesion Visualization Score
H *	6.374	5.330	4.303	4.926	7.321	6.076	4.978	5.392
*p*	0.041	0.070	0.116	0.085	0.026	0.048	0.083	0.068

FB, free-breathing; DIBH, deep-inspiration breath-hold; * Kruskal–Wallis H test was applied.

## Data Availability

The original contributions presented in this study are included in the article. Further inquiries can be directed to the corresponding authors.

## Abbreviations

The following abbreviations are used in this manuscript:

FB	Free-breathing
DIBH	Deep-inspiration breath-hold
BMI	Body mass index
ES	Edge sharpness
AP	Anteroposterior
SID	Source-to-image distance
IQR	Interquartile range

## References

[1] Laur O., Ha A.S., Bartolotta R.J., Avery R., Bateni C.P., Chen K.C., Dvorzhinskiy A., Flug J., Geannette C.S., Hinkle T. (2025). ACR Appropriateness Criteria^®^ Acute Shoulder Pain: 2024 Update. J. Am. Coll. Radiol..

[2] Kornguth P., Salazar A. (1987). The apical oblique view of the shoulder: Its usefulness in acute trauma. Am. J. Roentgenol..

[3] Chun L., Misaghi A., Cidambi K.R., McNeil N.P., Farnsworth C.L., Edmonds E.W. (2023). Validation of a Novel Radiographic View for Evaluating Proximal Humerus Fractures: The Clear View. J. Pediatr. Orthop. Soc. N. Am..

[4] Richardson J., Ramsay A., Davidson J., Kelly I. (1988). Radiographs in shoulder trauma. J. Bone Jt. Surgery. Br. Vol..

[5] Donnally C.J., Margetis K., Varacallo M.A. (2026). Vertebral Compression Fractures. StatPearls.

[6] Lança L., Franco L., Ahmed A., Harderwijk M., Marti C., Nasir S., Ndlovu J., Oliveira M., Santiago A.R., Hogg P. (2014). 10 kVp rule—An anthropomorphic pelvis phantom imaging study using a CR system: Impact on image quality and effective dose using AEC and manual mode. Radiography.

[7] Le N.T.T., Robinson J., Lewis S.J. (2015). Obese patients and radiography literature: What do we know about a big issue?. J. Med. Radiat. Sci..

[8] Manning-Stanley A.S., Ward A.J., England A. (2012). Options for radiation dose optimisation in pelvic digital radiography: A phantom study. Radiography.

[9] Doda Khera R., Singh R., Homayounieh F., Stone E., Redel T., Savage C.A., Stockton K., Shepard J.-A.O., Kalra M.K., Digumarthy S.R. (2019). Deploying Clinical Process Improvement Strategies to Reduce Motion Artifacts and Expiratory Phase Scanning in Chest CT. Sci. Rep..

[10] Yamamoto S., Hasebe T., Tomita K., Kamei S., Matsumoto T., Imai Y., Takahashi G., Kondo Y., Ito Y., Sakamaki F. (2020). Pulmonary perfusion by chest digital dynamic radiography: Comparison between breath-holding and deep-breathing acquisition. J. Appl. Clin. Med. Phys..

[11] Zhou W., Yin G., An J., Yang K., Teng F., Xu J., Bi X., Pang J., Chow K., Sirajuddin A. (2026). Clinical Feasibility, Efficiency, and Diagnostic Concordance of Full Free-breathing Cardiac MRI Compared with Breath-holding Techniques. Radiol. Cardiothorac. Imaging.

[12] Wang T., Li B., Han Q., Liao L., Mo R., Miao Y., Xu R., Zhang W. (2025). Effect of different breath-holding methods on scanning length and radiation dose of 320-row volumetric coronary CT angiography. Jpn. J. Radiol..

[13] Olthof S.-C., Reinert C., Nikolaou K., Pfannenberg C., Gatidis S., Benkert T., Küstner T., Krumm P. (2021). Detection of lung lesions in breath-hold VIBE and free-breathing Spiral VIBE MRI compared to CT. Insights Imaging.

[14] Sønderby A.H., Thomsen H., Skals R.G., Storm S., Leutscher P.D.C., Simony A. (2024). Thoracic spine X-ray examination of patients with back pain using different breathing technique and exposure times—A diagnostic study. Radiography.

[15] Lampignano J.P., Kendrick L.E., Bontrager K.L. (2020). Bontrager’s Textbook of Radiographic Positioning and Related Anatomy.

[16] Luo X., Chen G., Zhang S., Hu X., Huang C. (2025). Visualized breath-hold training for reducing respiratory motion artifacts in liver dynamic contrast-enhanced magnetic resonance imaging. Quant. Imaging Med. Surg..

[17] Dhiego Donizethe Ferreira G., Israel De Souza M. (2021). Evaluation of Chest X-Ray Quality Parameters. Int. J. Radiol. Imaging Technol..

[18] Allen C.M., Otto T.M. (2025). Clinical Preceptor Reference Guide for Student Competencies.

[19] Fujiwara Y., Muranaka Y. (2017). Improvement in visualization of carotid artery uniformity using silent magnetic resonance angiography. Radiol. Phys. Technol..

[20] Huda W. (2010). Review of Radiologic Physics.

[21] Mahesh M. (2013). The Essential Physics of Medical Imaging, Third Edition. Med. Phys..

[22] Steffensen C., Trypis G., Mander G.T.W., Munn Z. (2021). Optimisation of radiographic acquisition parameters for direct digital radiography: A systematic review. Radiography.

[23] Berrington De González A. (2009). Projected Cancer Risks From Computed Tomographic Scans Performed in the United States in 2007. Arch. Intern. Med..

[24] Mazars P., Garcia R. (2018). 12 Study of the anatomy position reproducibility during deep inspiration breath hold. Phys. Med..

